# Multiple site neural tube defects at Zewuditu Memorial Hospital, Addis Ababa, Ethiopia: a case report

**DOI:** 10.1186/s13256-021-02913-3

**Published:** 2021-08-17

**Authors:** Mestet Yibeltal Shiferaw, Atalel Fentahun Awedew, Tsegazeab Laeke T/Mariam, Abenezer Tirsit Aklilu, Yemisirach Bizuneh Akililu, Aemiro Mazengia Andualem

**Affiliations:** Neurosurgery Division, Department of Surgery, School of Medicine, Addis Ababa University, Addis Ababa, Ethiopia

**Keywords:** Case report, Ethiopia, Multiple neural tube defects, Neurulation, Re-zippering initiation model

## Abstract

**Background:**

Neural tube defects are common group of central nervous system anomalies of complex etiology and major public health importance worldwide. The occurrence of multiple neural tube defects, however, is an extremely rare event and has never been reported in Ethiopia so far. This study gives an insight into how the embryogenesis, management, and postoperative complications of multiple neural tube defects differ from the ordinary single neural tube defects on the basis of up-to-date existing literature.

**Case presentation:**

This paper highlights a case of an 8 days old female black race Ethiopian neonate who was brought by her mother with the chief complaint of lower back and lower neck swelling since birth. The findings were a 5 × 4 × 5 cm sized ulcerated placode at the mid-lumbosacral area and a 1.5 × 1.5 × 1 cm sized fluctuant, nontender, transilluminating mass with overlying unruptured defect dysplastic skin at the cervicothoracic junction. With a diagnosis of multiple neural tube defects secondary to unruptured cervicothoracic meningocele and ruptured lumbosacral myelomeningocele, single-stage repair of the defects was done with good outcome.

**Conclusion:**

There is insufficient evidence as to the exact mechanism of development of multiple neural tube defects. Similarly, whether patients with multiple neural tube defects had increased risk of post repair hydrocephalus compared with patients who have single neural tube defect is unknown. Hence, more research on the embryogenesis, management, and long-term outcome of multiple neural tube defects in particular and single neural tube defects in general should be done to better help patients with this costly and crippling problem. Lastly, the practice of folic acid supplementation is very low in resource-limited countries such as Ethiopia and, hence, should be improved.

## Background

Neural tube defects (NTDs) are common group of central nervous system anomalies that occur from multifactorial perturbation of normal neural development [1]. They are a major public health problem with significant morbidity, mortality, disability, and economic costs worldwide [2, 3]. The prevalence of NTDs varies across regions worldwide. According to a systemic review done in 2016, the median prevalence of NTDs was 11.7 per 10,000 births in Africa, 9 per 10,000 births in Europe, 11.5 per 10,000 births in America, and 21.9 per 10,000 births in Eastern Mediterranean [2]. A study conducted in the Addis Ababa and Amhara region, Ethiopia, revealed that NTDs were the leading cause of congenital anomalies and accounted for 40.3% of all congenital anomalies [4]. The prevalence of NTDs in Addis Ababa was 63.4 per 10,000 births and 126 per 10,000 births after 28 weeks and 12 weeks of gestation, respectively [5].

Multiple neural tube defects (MNTDs) are extremely rare congenital anomalies that are defined by the simultaneous occurrence of more than one NTD in a single case with “normal” neural tissue in between. It is mainly reported from developing African countries and India. It is a severe disorder that is thought to result from complex interaction of genetics and environmental factors, with folic acid deficiency being the most important environmental risk factor. There are different theories that explain how MNTDs and single NTDs develop, though the precise embryologic mechanism is still unknown [6].

## Case report

### History

An 8 days old female black race Ethiopian neonate born from a 28 years old para 1 abortion 1 mother by spontaneous vaginal delivery (SVD) at a gestational age of 40 + 1 week was brought by her mother to the pediatrics neurosurgical unit of Zewuditu Memorial Hospital with a complaint of lower back and lower neck swelling since birth. She had clear watery discharge from her lower back 1 day prior to her presentation. She was also not able to move her lower extremities since birth. Otherwise, she could suckle breast well and had no fever, abnormal body movements, or vomiting. Her mother lives in Addis Ababa and had unremarkable antenatal follow-up during her pregnancy. She, however, was not supplemented with folic acid before and during her pregnancy.

### Physical examination

**Fig. 1:**
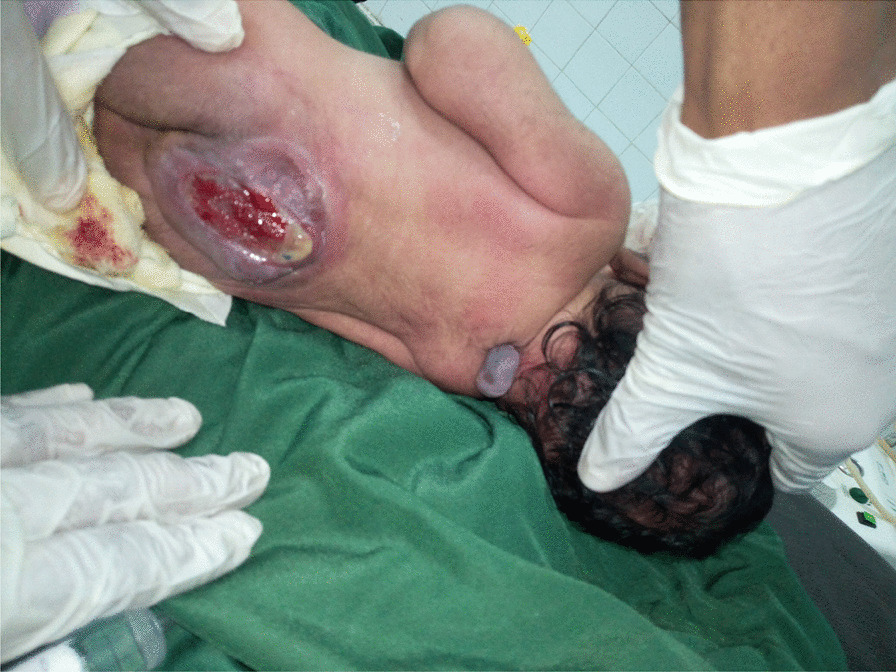
Cervicothoracic meningocele and lumbosacral ruptured myelomeningocele swellings (photo was taken after getting informed consent from the mother)

On physical examination, the baby was alert with normal vital signs. The baby had a 5 × 4 × 5 cm sized ruptured, shrunken and ulcerated placode at the mid-lumbosacral area. She also had a 1.5 × 1.5 × 1 cm sized fluctuant, nontender, transilluminating mass with overlying unruptured dysplastic skin at the mid-cervicothoracic junction area. She had lower limb paralysis. The head circumference was 31 cm, which lies within the normal range for her age (Fig. 1).

### Investigations

Her laboratory results were all in the normal range but did not include imaging because of an affordability issue.

### Diagnosis

Based on the clinical examination findings, the patient was clearly diagnosed to have multiple neural tube defects secondary to ruptured lumbosacral myelomeningocele and unruptured cervicothoracic meningocele.

### Surgical procedure

After informed written consent was obtained, the patient was taken to operation room. The patient was intubated supine and repositioned to prone position. The surgical field was then cleaned with antiseptics (both alcohol and providone) and draped properly. Lidocaine with adrenaline was administered at the defect sites to minimize intraoperative bleeding and decrease postoperation pain. First, incision was made at the junction of the dysplastic and normal skin of the lumbosacral defect to free the placode. There was fibrinous material but no gush of cerebrospinal fluid in doing so. The fibrinous material was removed gently. The placode was then tubularized to facilitate optimal skin closure and decrease postoperation retethering. Nerve roots were freed from the arachnoids and dura circumferentially dissected. Hemostasis was secured with bipolar cautery and dura closed with proline (6, 0) in watertight fashion. Absence of cerebrospinal fluid (CSF) leak was checked using Valsalva maneuver. Repair of cervicothoracic meningocele was done using a similar technique. There was gush of clear CSF upon opening of the dysplastic skin, and the sac had communication with subdural space of the spinal cord. Part of the meninges sac was then excised and closed with proline (6, 0) in watertight fashion. Subcutaneous tissue and skin was also closed using vicryl (3, 0) at both sites. Patient was extubated and transferred to postanesthesia care unit with stable vital signs. Single-stage repair of the defects was completed safely.

### Postoperative course and follow-up

Postoperatively, the patient’s clinical status including serial head circumference was followed. Accordingly, she had no repair site CSF leak, wound failure, meningitis, or postrepair hydrocephalus. She took antibiotics for the ruptured myelomeningocele for 7 days. On her seventh postoperative day, she was discharged with no complication. Currently, the patient is on her 11th smooth postoperative month on our follow-up, requiring no shunt so far.

## Discussion

Among the 57 MNTDs that existed in published literatures, only 3 were MNTDs that consisted of cervicothoracic and lumbosacral myelomeningocele [6], ours being the fourth case (6.9% of MNTDs were cervicothoracic and lumbosacral myelomeningocele). Multiple site neural tube defects (MNTDs) were never reported in Ethiopia, probably because of poor documentation and reporting, despite single-site NTD being the most common congenital anomaly in Ethiopia [4, 5]. Proper documentation of this case was possible as initial evaluation of the patient’s clinical presentation, therapeutic interventions, and postoperative follow-up were given by authors, while delayed presentation to our neurosurgical unit was the pitfall in the management of this patient.

The exact embryologic mechanism of how single- and multiple-site NTDs occur continues to be an area of controversy. To date, there exist three major hypotheses in this regard. These are the “single site” closure theory also known as zipper-like closure theory, the multisite closure theory, and re-zippering initiation model [7, 8].

The more widely accepted zipper-like closure (“single site” closure) theory explains the occurrence of single neural tube defect, and it states that the neural tube closure begins in the mid-cervical region and then progresses to close the caudal and cranial neuropores (on days 24 and 26, respectively) in a zipper-like fashion. According to this theory, the most caudal or the most rostral end should have been the most common site of an myelomeningiocele (MMC) [6]. It, however, fails to explain how MNTDs and cranial NTDs do occur and why some types of NTDs are more common in some regions of the craniospinal axis. The areas caudal to S-2 form through secondary neurulation due to the neural tube forming above S-2, a concept that the three theories agree on.

**Fig. 2:**
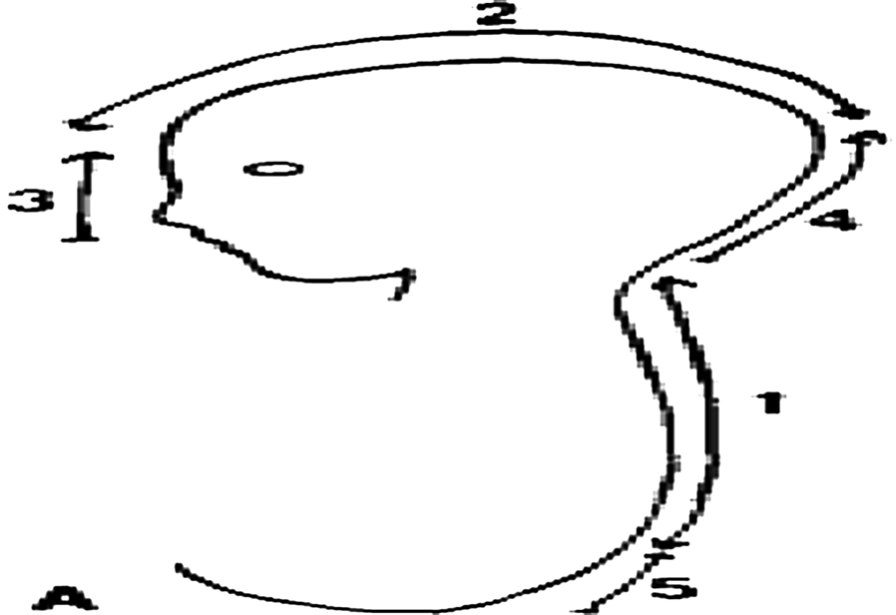
Multisite closure model of neural tube ( Adapted from Van Allen *et al*. 1993)

The “Multiple initiation sites of closure model,” which was initially proposed by Van Allen *et al*., hypothesizes that there are five closure sites and that closure proceeds in an orderly fashion. Initially, it begins at site 4, followed by sites 2 and 3 and then sites 1 and 5. This theory tries to explain how the development of MNTDs occurs. However, it is subject to criticism because of the rarity of MNTDs and continuous debate on the exact number of multiple sites of neural tube closure [7] (Fig. 2).

**Fig. 3:**
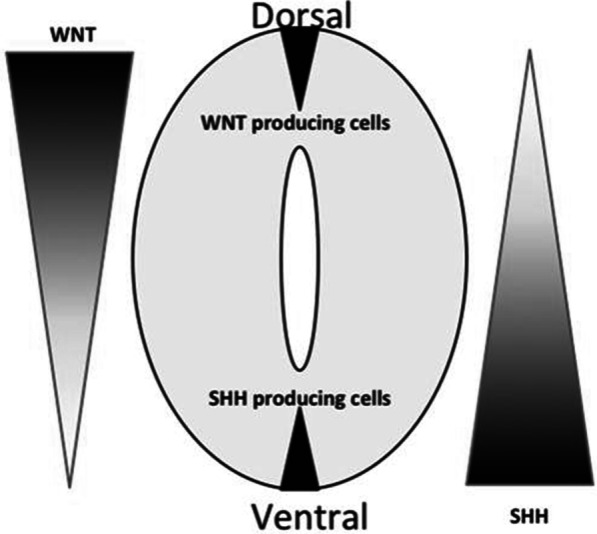
*Shh* and *Wnt* interactions for ventral and dorsal neural tube closure, the key concept in re-zippering model of neural tube closure ( Adapted from T.W Vogel *et al*. 2012)

The “re-zippering initiation model” from interrupted site of closure, proposed by Mahalik *et al*., hypothesizes that it is the interplay of *Shh* (Sonic hedgehog) and its antagonist *Wnt* that mediates the initiation and sustenance of neural tube closure. Normally, the antagonistic interactions between canonical *Wnt*, promoting dorsal identities, and *Shh* pathways, inducing ventral ones, would define the dorsoventral patterning of the developing central nervous system. In other words, *Shh* inhibits dorsolateral hinge point formation, and *Wnt* represses *Shh* activity. Mahalik *et al*. have stated that the focal alterations in the magnitude of *Shh* signaling lead to focal disruption of neural tube closure, while the normal neural tube closes normally in the adjacent segments. Thus, each segment will have an independent potential to close. Any local environmental insult to these regulatory pathways can affect the ability of succeeding segments to close, while the duration and local extent of the insult determine the extent of NTD. Once the causative insult subsides, the subsequent segments close because of their innate potential (Fig. 3).

The author gives emphasis to this theory as it better explains how any of the neural tube defects including MNTDs come into existence by taking the strong sides of previous theories and addressing their limitations. It also uses the results of many developmental neurobiology studies that explain the neural tube closure much better than the previous theories [8].

The MNTDs can be repaired with a single- or multiple-staged procedure. Whether to repair the MNTDs as a single- versus multiple-staged procedure is mainly a function of patient’s tolerance to the duration of anesthesia and the anticipated blood loss for patient’s age. Accordingly, single stage repair of defects is safe and not associated with increased postoperative complications. The repair for our case was done as a single-staged procedure with smooth postoperative course, which is consistent with other case reports [9]. However, whether patients with MNTD had increased risk of post-defect-repair hydrocephalus compared with single NTD patients is unknown. Hence, further study is needed to guide clinicians’ decision in management.

### Prevention of neural tube defects

Folic acid deficiency among women of reproductive age is > 20% in most low-income countries while < 5% in high-income countries according to a systematic review done to assess global folic acid deficiency in women of reproductive age [10]. A public health importance of folic acid is defined by folic acid deficiency prevalence of > 5% among women of reproductive age. In Ethiopia, only one-third (32.7%) of reproductive women had normal (> 6.6 ng/mL) serum folic acid level, while nearly half (46.1%) of them had severe (< 4 ng/mL) deficiency [11].

Evidence from randomized control trials (RCT) clearly revealed that supplementation of folic acid could have decreased NTD recurrence rate by 72% [12, 13]. The practice of folic acid supplementation should be emphasized in resource-limited countries, including Ethiopia, as only 13.5% of mothers having kids with NTD get folic acid supplementation compared with 30.6% of mothers who deliver normal babies [5].

## Conclusion

There is insufficient evidence as to the exact mechanism of MNTD development. Similarly, whether patients with MNTD had increased risk of postrepair hydrocephalus compared with single neural tube defect remains unknown. Hence, more research on the embryogenesis, management, and long-term outcome of MNTDs in particular and single NTDs in general should be done to better help patients with this costly and crippling problem. Lastly, the practice of folic acid supplementation is very low in resource-limited countries such as Ethiopia and should be improved.

## Data Availability

Availability of supporting data: besides the patient’s picture, the photo of the patient’s chart is available from corresponding author and can be shared when needed.

## Abbreviations

CSF: Cerebrospinal fluid
MNTD: Multiple neural tube defects
NTDs: Neural tube defects
RCT: Randomized control trial
SVD: Spontaneous vaginal delivery
